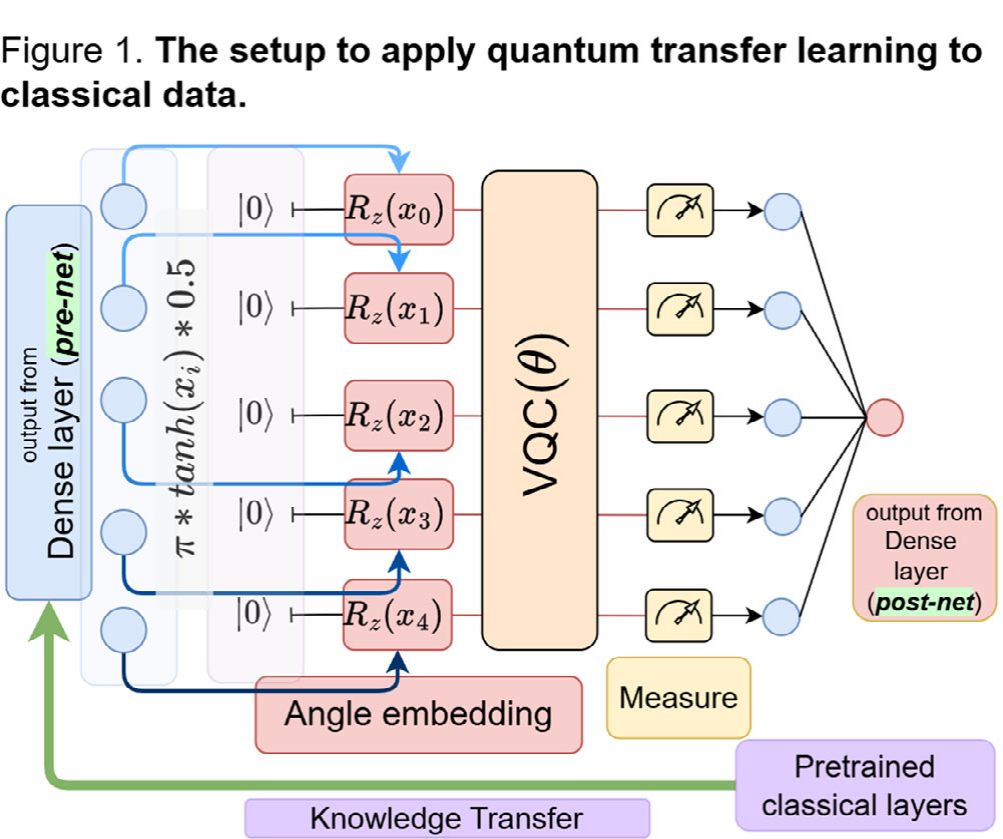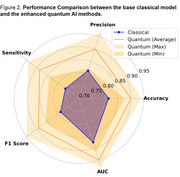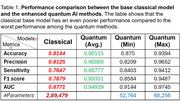# Quantum AI Based Enhanced Detection of Dementia

**DOI:** 10.1002/alz70856_103249

**Published:** 2025-12-25

**Authors:** Sounak Bhowmik, Talita Perciano, Himanshu Thapliyal

**Affiliations:** ^1^ University of Tennessee, Knoxville, TN, USA; ^2^ Lawrence Berkeley National Laboratory, Berkeley, CA, USA

## Abstract

**Background:**

Quantum computing has the potential to significantly improve the early detection of Alzheimer's Disease and Related Dementias (ADRD). Quantum‐enhanced machine learning can be used to perform an early screening of Alzheimer's disease using brain imaging data based on dataset of MRI scans from both healthy individuals and those diagnosed with Alzheimer's. This study aims to demonstrate the potential of quantum transfer learning to enhance the performance of the classical deep learning model for dementia detection.

**Method:**

Using the MRI sagittal images available in the OASIS‐2 (64 demented and 72 non‐demented subjects between 60 and 96 years), we show how quantum techniques can transform a suboptimal classical model into a more effective solution for dementia detection, highlighting their potential impact on advancing healthcare technology. We begin with a simple classical deep learning model with a significantly smaller number of parameters, which gives suboptimal performance on the problem. Then, we apply different configurations of quantum transfer learning based on the pre‐trained weak classifier (Figure 1). We fix the weak classifier's initial convolutional layers at their fixed pre‐trained parameters and replace the last set of dense layers with a dressed quantum circuit (DQN), which we train to enhance performance. We performed 4‐fold cross‐validation for both the classical and the hybrid quantum models and trained them using Pennylane's `default.qubit' simulator and IonQ's Aria‐1 simulator (noisy simulation).

**Result:**

We showed that with significantly fewer parameters, the quantum transfer learning‐based hybrid models showed significant performance enhancement over the base weak classical deep learning model for dementia detection. To classify between a demented and non‐demented subject, the accuracy of quantum‐based AI methods improved by 6 to 14% compared to classical methods. The sensitivity of the models improved by 4 to 17%. This shows that there are fewer chances of misclassifying demented patients. Figure 2 compares the performance of the hybrid quantum models and their base classical model, and Table 1 summarizes the results.

**Conclusion:**

We illustrated that with assistance from quantum machine learning, it is possible to enhance detection for dementia based on brain images. This shows the potential for practical utility of quantum computing in ADRD research.